# Performing oncological procedures during COVID-19 outbreak: a picture from an Italian cancer center

**DOI:** 10.37349/etat.2021.00058

**Published:** 2021-10-31

**Authors:** Maristella Bungaro, Valentina Bertaglia, Marco Audisio, Elena Parlagreco, Chiara Pisano, Valeria Cetoretta, Irene Persano, Francesca Jacobs, Chiara Baratelli, Lorena Consito, Maria Lucia Reale, Fabrizio Tabbò, Paolo Bironzo, Giorgio Vittorio Scagliotti, Silvia Novello

**Affiliations:** Medical Oncology, San Luigi Gonzaga University Hospital, University of Torino, 10043 Orbassano, Italy; Università Politecnica Marche, Italy

**Keywords:** COVID-19, prevention, cancer care, diagnosis, oncological procedures

## Abstract

**Aim:**

Since SARS-CoV-2 infection rapidly spread around the world, Italy has quickly become one of the most affected countries. Healthcare systems introduced strict infection control measures to ensure optimal care, especially in frail groups such as cancer patients (pts). This study investigated the efficacy of SARS-CoV-2 pre-procedure screening and whether COVID-19 influenced timely diagnosis and therapy.

**Methods:**

Data of oncological procedures of pts with confirmed or suspected cancer diagnosis, treated at Oncology Department or coming from Emergency Department of San Luigi Gonzaga Hospital between June 2020 and March 2021 were retrospectively collected. A nasopharyngeal swab (NPS) was performed in outpatients 24/48 h before procedures. Inpatients were tested by NPS before and after hospitalization.

**Results:**

Two hundred and twenty-one pts were included in this analysis. Median age was 73 years, males were 58%. Eastern Cooperative Oncology Group (ECOG) Performance Status was 0 or 1 in 88% of pts. The most frequent cancer type was lung cancer (57%). Stages IV were 77%. Two hundred and forty-three scheduled procedures were performed with diagnostic (*n*: 142; 58%), therapeutic (*n*: 55; 23%), and palliative (*n*: 46; 19%) intent. One hundred and four and 139 procedures were performed in out- and in-pts, respectively. Of the 234 NPS performed, 10 (4%) were positive. Two pts were infected during hospitalization, 8 in community. Most of them were asymptomatic, while only 2 had mild symptoms. Eight procedures (3%) were postponed, 1 cancelled, while 2 were performed in positive pts. Median time to resolution of the infection was 17 days (11–36). Median delay in the procedures was 25 days (14–55). Five pts started systemic treatment, after a median time of 37.5 days (13–57).

**Conclusions:**

SARS-CoV-2 infection led to the postponement of a small, but not negligible percentage of oncological procedures. However, the low infection rate observed suggests that structured screening for COVID-19 is critical for the best management of scheduled procedures during pandemic.

## Introduction

At the end of 2019, severe acute respiratory syndrome coronavirus 2 (SARS-CoV-2), was first reported in Wuhan, China, and rapidly spread across the globe. On 11th March, 2020, the World Health Organization declared coronavirus disease 2019 (COVID-19) a pandemic. Since then, Italy has quickly become one of the worst affected countries, with a reported case number of around 2 million, and 76,000 deaths in December 2020 [1].

Patients (Pts) with cancer were considered to be at high risk of SARS-CoV-2 infection due to both low immunity and the need for regular hospital access for cancer treatment [2, 3]. Most importantly, cancer pts were observed to have an increased risk of serious clinical complications compared with pts without cancer [4].

In this challenging situation, non-urgent health services have been suspended, while health facilities have made efforts to ensure pts have access to diagnostic, therapeutic and palliative cancer services. In order to prevent the transmission and ensure optimal care, healthcare systems modified their practice by introducing strict infection control measures for hospital access [5–7].

Starting from June 2020, after the first wave of contagions in Italy, the Oncology Unit of San Luigi Gonzaga University Hospital implemented a COVID-19 screening protocol for candidates to perform oncological procedures. This study aimed to investigate the efficacy of pre-procedure screening for COVID-19 and whether infection influenced the opportunity of pts to receive timely diagnosis and therapy.

## Materials and methods

Retrospective data of oncological procedures performed at San Luigi Gonzaga University Hospital between June 2020 and March 2021 were collected using electronic medical records. According to National Health Authorities measures, the first pandemic wave started in February 2020 and ended in June 2020, and the second wave from October 2020 to January 2021, while the third wave from February 2021 to April 2021 [1]. The time period considered in this study includes recovery from the first wave, lockdown and recovery from the second wave and lockdown of the third wave. General population vaccination started in February 2021.

Pts included in this analysis had a confirmed or suspected diagnosis of cancer and were being treated as outpatients in the Department of Oncology or hospitalized after accessing the Emergency Department (ED).

**Figure 1.**
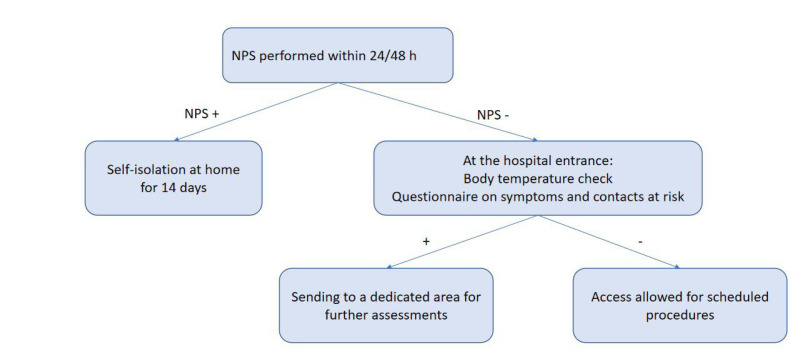
Flow chart of the COVID-19 screening protocol for outpatients

**Figure 2.**
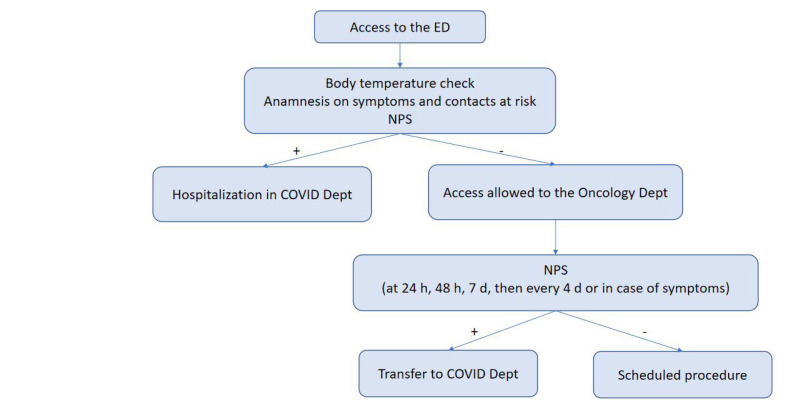
Flow chart of the COVID-19 screening protocol for inpatients. Dept: Department; h: hours; d: days

According to our internal COVID-19 protocol, outpatients underwent body temperature check upon entering the hospital. They were also subjected to a questionnaire that investigated symptoms suggestive of infection (fever, cough, dyspnea and diarrhea) and any contacts with positive subjects in previous days. Pts with suspicious findings were referred to a dedicated area for further examinations. Outpatients received molecular nasopharyngeal swab (NPS) within 24/48 h before oncological procedures (Figure 1). Inpatients were tested by NPS before hospitalization, after 24h, 48 h, seven days and then every four days or in case of appearance of symptoms (Figure 2). All swabs were performed by dedicated nurses and analyzed by VIASURE^®^ SARS-CoV-2 S gene Real Time PCR (Polymerase Chain Reaction) Detection kit at the San Luigi Gonzaga hospital laboratory. The kit has a detection limit of ≥ 10 copies of RNA per reaction for SARS-CoV-2. Descriptive statistics were used to summarize data. Categorial variables were summarized as counts and percentages.

## Results

Two hundred and twenty-one pts were included in this analysis. Median age was 73 years (range 22–90). Males were 128 (58%), while females were 93 (42%). Most pts were in good clinical conditions (88%), with Eastern Cooperative Oncology Group (ECOG) Performance Status (PS) of 0 or 1 (PS 0 in 87 pts, PS 1 in 109, PS 2 in 24 and PS 3 in 1 patient). The most frequent cancer type was lung cancer (127 pts, 57%). Other types of malignancies were gastrointestinal (56 pts, 25%), neuroendocrine (9 pts, 4%), genitourinary (8 pts, 4%), gynecological (6 pts, 3%), head and neck tumors (2 pts, 1%), sarcomas (3 pts, 1%), breast cancer (10 pts, 5%). Fifty (23%) pts had early disease, while 171 (77%) had advanced stages. Patient characteristics are summarized in Table 1. Two hundred and forty-three scheduled procedures were performed: 101 biopsies, 43 bronchoscopies, 7 surgeries, 1 trans-arterial radioembolization, 1 trans-arterial chemoembolization, 22 radiofrequency ablation (RFA), 15 microwave ablation, 1 cryoablation, 5 paracenteses, 5 thoracentesis, 7 gastroscopies, 1 endoscopic ultrasonography (EUS), 6 endoscopic retrograde cholangiopancreatographies, and 3 biliary drainages. One hundred and forty-two (58%) procedures were diagnostic, 55 (23%) therapeutic and 46 (19%) palliative. One hundred and four (43%) and one hundred and thirty-nine (57%) procedures were performed in out and in-pts, respectively. Two hundred and thirty-four NPS were performed prior to scheduled procedures, 10 of which (4%) were found to be positive. All positive pts were infected during the second wave. Two pts were infected during hospitalization, while 8 in community. Most of them were asymptomatic (8 pts), while the others had mild symptoms (mainly fever). Eight procedures (3%) were postponed (6 diagnostic, 1 therapeutic and 1 palliative): 1 EUS, 3 biopsies, 1 thoracoscopy, 1 RFA, and 2 bronchoscopies. An explorative bronchoscopy was canceled, while a diagnostic biopsy and an abscess drainage were performed even though the pts tested positive. Median time to resolution of the infection was 17 days (range 11–36). Median delay in the procedures was 25 days (range 14–55). Five pts started systemic treatment in a median time of 37.5 days (range 13–57); a patient underwent therapeutic RFA on the same day as the biopsy, and two pts continued the ongoing treatment. No COVID-related deaths occurred. The main results are summarized in Table 2.

**Table 1.** Summary of the main characteristics of the pts

**Characteristic**		**Value**
Total number, *n.*		221
Males / Females, *n.* (%)		128 (58) / 93 (42)
Median age		73 (22–90)
Tumor type, *n*. (%)	Lung and chest	127 (57)
	Gastro-intestinal	56 (25)
	Breast	10 (5)
	Genito-urinary	8 (4)
	Neuroendocrine	9 (4)
	Gynecological	6 (3)
	Sarcomas	3 (1)
	Head and neck	2 (1)
Stage, *n*. (%)	Early	50 (23)
	Advanced	171 (77)
ECOG PS, *n*. (%)	0	87 (39)
	1	109 (49)
	2	24 (11)
	3	1 (< 1)

*n*.: number

**Table 2.** Summary of scheduled procedures, swabs performed and main results

**Parameter**	**Value**
Total number of procedures, *n.*	243
Diagnostic, *n.* (%)	142 (58)
Therapeutic, *n.* (%)	55 (23)
Palliative, *n.* (%)	46 (19)
Inpatients / Outpatients, *n*. (%)	139 (57) / 104 (43)
Total number of NPS performed, *n*.	234
Positive NPS, *n*. (%)	10 (4)
Negative NPS, *n*. (%)	224 (96)
Postponed procedures, *n*. (%)	8 (3)
Cancelled procedures, *n*. (%)	1 (0.7)
Median delay of the postponed procedures, days (range)	25 (14–55)

## Discussion

This work describes the protocol established by the Department of Oncology of the San Luigi Gonzaga Hospital in agreement with the hospital management to avoid the spread of the coronavirus disease among pts and between pts and healthcare workers. The aim of the study was to investigate the efficacy of pre-procedure screening for COVID-19 and whether infection affected the opportunity of pts to receive timely diagnosis and therapy.

During pandemic, 243 scheduled procedures were performed, mainly lung biopsies, bronchoscopies, endoscopic diagnoses of the gastrointestinal tract and loco-regional treatments of liver lesions. Pts included in this analysis had a confirmed or suspected diagnosis of cancer, for which they were being treated as outpatients in the Department of Oncology or hospitalized after an access to the ED for acute clinical conditions. 43% and 57% of the procedures were performed in outpatients and hospitalized pts, respectively. Notably, this study includes a high number of pts affected by lung cancer, probably because the center where it was conducted is of referral for thoracic malignancies.

According to the internal COVID-19 protocol, NPS was performed in outpatients within 24–48 h before oncological procedures. Following this protocol, only eight procedures (3%) were postponed and one canceled due to NPS positivity. Two procedures were performed even if pts tested positive.

These two procedures were a drainage of a post-surgical retroperitoneal abscess and a diagnostic lymph node biopsy, with one performing during hospitalization and the other on outpatient, respectively. In the second case, after the biopsy was executed, the patient underwent a 21-day period of self-isolation at home, as established by the national health authorities. This last case underlines how the management of COVID-positive pts with cancer is still unclear, as contagion risk of healthcare workers performing procedures with protective equipment might be low and long term molecular NPS positivity may not be associated with contagiousness. Indeed, other clinical reports on diagnostic and interventional procedures on COVID-19 positive pts with cancer have been already reported, supporting our data [8, 9].

Considering these data, we can assert that our clinical activity continued without major hitches despite the emergency. Only a small percentage of the scheduled oncological procedures have been delayed, although this finding is not negligible. Screening pts for SARS-CoV-2 infection before accessing interventional radiology and endoscopy services has been helpful in avoiding the spread of the virus in the hospital. Of the pts found positive, most had few symptoms and were able to perform the scheduled procedure with a maximum delay of 55 days (range 14–55). Indeed, we did not record any deaths from COVID-19 in our cohort.

The management of cancer pts during COVID-19 pandemic is very challenging, given their known condition of frailty, the high risk of infection and the need for regular access to hospitals to receive treatment [10]. According to the study by Liang and colleagues [4], cancer patient had a higher risk of developing serious COVID related events, defined as the need of invasive ventilation in intensive care unit or death, compared with general population (39% *vs.* 8%; *P* = 0.0003). The risk of serious events appeared to be higher in pts who had received chemotherapy or surgery in the previous month [4]. In addition, worse outcomes have been reported for cancer pts suffering from SARS-CoV-2 infection: the Chinese Center for Disease Control and Prevention reported that the case mortality rate was higher among subjects with comorbid conditions (5.6% for cancer pts, compared with 2.3% in the overall population) [11]. To face this emergency situation, oncology societies and national authorities quickly released international guidelines on the management of cancer care during COVID-19 pandemic [12]. General recommendations included the reduction of clinical visits, the preference for telemedicine when applicable, the establishment of priority groups for surgery, systemic treatments and radiotherapy [12].

The management of oncological procedures has certainly required great efforts, in line with what is happening in the rest of the world.

According to the experiences reported by Italian and foreign centers, the management of pts with chronic non-oncological diseases was also very difficult during the pandemic.

For example, elective and non-urgent visits at the Neuromuscular Disorder Center in Milan, Italy, were postponed or conducted on virtual platforms. Diagnostic services were suspended, except for urgent cases of amyotrophic lateral sclerosis, myasthenia, myositis or immune-mediated neuropathies. Lung assessments were stopped to avoid the risk of viral spread from pts to staff members. Tests for dysphagia were performed only for pts at high risk of complications [13].

Telemedicine, in the form of virtual visits and phone consultations, has played a critical role also in the management of pts affected by diabetes and obesity, as reported by an international virtual meeting that took place on August 2020 among experts from South and East Europe (including Italy), Middle East and Africa [14].

Hospital access also dropped down due to users’ fear of contagion too. A research network of Italian pediatric hospitals reported delayed access to ED in 12 pts with symptoms simulating COVID-19 that turned out to be related to life-threatening conditions, including diabetic ketoacidosis, acute-onset leukemia, bacterial pneumonia and pyelonephritis [15].

A protocol to regulate hospital access similar to ours was used at the Center for Hematology and Oncology in Marrakesh, Morocco, where a screening zone was organized for any patient entering the center. The screening zone received approximately 100 pts per day. Subjects with suspected symptoms of contagion were referred to COVID investigative facilities to perform NPS. Only 8 pts showed symptoms of fever and cough, but were negative on NPS testing for SARS-CoV-2. Moroccan colleagues reported a 0% infection rate among health care workers [16].

Based on Ontario (Canada) provincial Ministry of Health guidelines recommending baseline COVID-19 testing for cancer pts before receiving immunosuppressive cancer treatment, Madariaga and colleagues [17] proposed a model for Sars-CoV-2 testing in asymptomatic pts. The authors proposed to test pts by real-time reverse transcription PCR before initiating systemic therapy, radiation and/or surgery, especially those with high-risk characteristics, such as advanced age, ECOG PS ≥ 2, significant comorbidities and lymphopenia.

A prospective observational study on the role of routine screening (RS) for SARS-CoV-2 infection in cancer pts undergoing active cancer-directed therapies (CDT) was conducted at the Mayo Clinic Cancer Center. A screening protocol was adopted to identify cancer pts with COVID-19 before initiating CDT and before every subsequent cycle of systemic therapy. The aim was preventing complications related to concomitant exposure to COVID-19 and CDT. The results were recently published [18]. Xie and colleagues compared the outcomes of the cohort of cancer pts who were diagnosed with COVID-19 by RS with those diagnosed on the basis of clinical suspicion or history of exposure (nonroutine screening, NRS). They found that COVID-19 positivity rate was low on the basis of RS (0.81%). Comparing the hospitalization rate, intensive care unit admission and mortality, there were no significant differences between the RS and NRS group [18].

Ensuring the continuity of care for cancer pts is one of the biggest challenge clinicians faced during the pandemic [19, 20]. Many of the early detection and prevention programs have been suspended or delayed [21, 22]. The limitation of hospital visits in order to avoid contagion for cancer pts has led in some cases to postpone primary diagnosis and delay access to treatments [21]. Diagnostic delay may impact pts’ survival due to the so called “stage migration” towards advanced disease [23]. At the same time, access to health services and exposure to a potential SARS-CoV2 infection can be very risky for pts with active cancer.

The National Screening Observatory monitored the performance of regional screening programs for cervical, breast and colon cancer during the coronavirus epidemic in Italy. Surveys conducted showed a decrease in the propensity to participate in screening in 2020 compared to 2019, less pronounced for cervical and mammography screening (–15%), higher for colorectal screening (–20%). The average diagnostic delay is getting longer and amounts to 5.5 months for colorectal lesions, 4.5 months for breast cancer and 5.2 months for cervical lesions [24].

Our efforts must be aimed at balancing the need to implement measures to contain the transmission of the virus with the need for pts to receive adequate care.

This analysis has some limitations. First, the study was conducted in a single center, and therefore our findings are not necessarily applicable to other Italian regions, where health resources are different and the rate of spread of infection is highly variable. Second, due to the small sample size, comparisons across different cancer types were not made. The third limitation is the retrospective nature of the study. Moreover, no data was available to compare these results with the clinical activity of our center in the pre-COVID era. Despite these limitations, this work suggests that cancer care can continue safely during the pandemic.

In conclusion, COVID-19 disrupted and reshaped the practice of oncology worldwide. Much emphasis was placed on the vulnerability of the cancer patient to the infection, the consequences of contagion and the implications of reduced health services. Despite the difficulties brought about by the pandemic, our center continued its clinical activity smoothly. Our main achievement has been to develop a suitable strategy to protect our pts and healthcare professionals from contracting SARS-CoV-2 without affecting the quality of our healthcare.

## Abbreviations

CDT: cancer-directed therapies
COVID-19: coronavirus disease 2019
ECOG: Eastern Cooperative Oncology Group
ED: Emergency Department
NPS: nasopharyngeal swab
PS: Performance Status
pts: patients
RFA: radiofrequency ablation
SARS-CoV-2: severe acute respiratory syndrome coronavirus 2

## Declarations

### Copyright

© The Author(s) 2021.
